# “ABC”—The Awareness-Body-Chart: A new tool assessing body awareness

**DOI:** 10.1371/journal.pone.0186597

**Published:** 2017-10-16

**Authors:** Ursula Danner, Alexander Avian, Tanja Macheiner, Beate Salchinger, Nina Dalkner, Frederike T. Fellendorf, Armin Birner, Susanne A. Bengesser, Martina Platzer, Hans-Peter Kapfhammer, Michel Probst, Eva Z. Reininghaus

**Affiliations:** 1 Department Health Sciences/Physiotherapy, FH Campus Wien, University of Applied Sciences Vienna, Vienna, Austria; 2 Department of Psychiatry and Psychotherapeutic Medicine, Medical University of Graz, Graz, Austria; 3 FH JOANNEUM Graz, University of Applied Sciences, Graz, Austria; 4 Institute for Medical Informatics, Statistics and Documentation, Medical University of Graz, Graz, Austria; 5 Department of Rehabilitations Sciences, KU Leuven-University of Leuven, Leuven, Belgium; Monash University, AUSTRALIA

## Abstract

**Background:**

Despite the importance of body awareness for health and well-being there is still a lack of valid assessment tools to scan proper body awareness. To respond to the limitations of questionnaires (reading/interpretation problems) the Awareness-Body-Chart (ABC) was designed to assess body awareness by colouring 51 regions according to their awareness. The objective of this study was to investigate the psychometric characteristics of the ABC.

**Methods:**

In a questionnaire-study, 106 students in Graz (79 females, 27 males, age median 21 (*IQR* 20–23) years) filled in the ABC, furthermore a German body awareness questionnaire „KEKS”, and the Beck Depression Inventory II. Factor structure, internal consistency, and retest reliability of the ABC were investigated. Correlations of the ABC with the KEKS and the Beck Depression Inventory II and comparisons of subgroups were conducted.

**Results:**

Through factor analyses, 14 factors with clear assignments to body parts could be categorized: cranium, face, cervical/lumbar region, chest/abdomen, back, shoulder, upper arm, lower arm/elbow, hand, genital area, thigh/hip, knee, lower leg, and foot. The 14 body parts and the total score showed acceptable to high Cronbach’s alphas (*α* = .64 - .97). The test-retest reliability showed values between *ρ* = .71 and *ρ* = .96. The correlation of the ABC and KEKS (*r* = .66, *p* < .001) confirmed validity. Further indications of validity could be seen in comparisons of subgroups and in correlations with the Beck Depression Inventory II.

**Conclusion:**

The ABC proved good psychometric properties with acceptable to high internal consistency, acceptable to high retest reliability and high construct validity. It is an easy-to-use tool for clinical settings and research. The ABC opens new insights into body awareness-patterns of various subgroups.

## Introduction

Though there are various concepts and subconcepts of body awareness and a confusion of definitions around the term of body perception in the literature [1–3], there is growing evidence for the importance of body awareness–here defined as the subjective experience of one’s body and the particular body parts—for physical stability and wellbeing [4–7]. Body awareness can be seen as the transition from objective to subjective sensory physiology [8]. It is the interface between physiological body-perception (i.e. visual, tactile, olfactoric, gustatoric, auditive or kinaesthetic as well as visceral perception) and cognitive-affective processing in the nervous system [9]. As a consequence, vigilance and concentration have a big influence on the processing of stimuli [9]. Furthermore, body awareness is modified by mental processes including attitudes and affects, interpretation, appraisal, beliefs, cultural imprint, memories and conditioning. These aspects are also responsible for the evolution of one’s body image which always interacts with the individual body awareness [2, 10]. Body awareness is seen as a basic dimension in the evolution and the concept of the body, as well as the related emotional and affective dynamics [11]. Body awareness is often considered on a subconscious level and is distinguishable from and interacts with thoughts, affective states and exteroceptive stimuli.

In English literature, the term interoception has gained popularity in research around the perception of the state of the body. Interoception may be described as the sensation concerning the state of the internal body and the internal organs, distinguished from proprioception as the reflection of the body in space and exteroception as the perception of the external environment [12]. In the last years, there has been a surge in theoretical and empirical work on the interoceptive system (like the feeling of temperature and pain) as described by Craig et al. [13]. Interoception is supposed to be associated with autonomic motor control—in distinction from the exteroceptive systems (cutaneous mechanoreception and proprioception) that guide somatic motor activity [14]. According to Craig et al., in humans the neural pathway of interoception leads to the anterior insula of the limbic sensory cortex which seems to provide the basis for the subjective image of the emotional awareness, the “self as a feeling entity” [6]. In literature, we find growing interest in the neuroanatomical pathways and processes of interoception concerning concepts of pain, of the embodied cognition, results of improved memory and improved decision-making by enhanced interoceptive accuracy (i.e. performance on objective behavioural tests of heartbeat detection) [12]. Furthermore, altered interoceptive states were found in psychiatric disorders as e.g. in anxiety and depression [15]. Recently Garfinkel et al. [12] describe a lack of correspondence between interoceptive accuracy and interoceptive sensibility (self-evaluated assessment of subjective interoception) and interoceptive awareness (metacognitive awareness of interoceptive accuracy). In a study by Khalsa et al. [16] the insula turns out not to be the sole necessary substrate for interoceptive awareness. The authors demand a comprehensive redefinition of the concept of interoception involving “afferent information that arises from anywhere and everywhere within the body” including perceptions through the skin via pathways usually considered to support exteroception [16]. In a review by Ceunen et al. [3], the origins and developments of the concept of interoception from the very first use of the word interoception in a publication—by Sherrington in 1906 [17]—to actual scientific concepts of interoception are summarized. The authors clarify that the definition of interoception in literature ranges from restrictive to very inclusive meanings. In the restrictive meaning only sensations from the viscera are interoceptive. More common in recent literature is the conception of an inclusive sense of interoception, regardless of what information the brain uses and does not use to construct its perception of the state of the body. Additionally, Garfinkel et al. [12] distinguish subjective, objective and metacognitive aspects of perception. The subjective dimension of interoception interpreted in the inclusive way, is congruent to our definition of body awareness—a subjective interpretation of the body state.

Assessing body awareness is a complex multidimensional challenge on physiological and psychological levels and always remains a compromise of objective information and subjective interpretation [18, 19]. There are many strategies used to assess the body-concept of a person [20], but the phenomenon of body awareness itself has not been considered to any great extent. Existing assessments have a strong psychological orientation or are aimed at assessing a specific bodily function as e.g. heartbeat tracking task [21]. In German literature only a few tools are validated for the investigation of body awareness. In the book „Körpererleben und Körperbild–ein Handbuch zur Diagnostik”[2] the following questionnaires are presented: „Der Fragebogen zum Körperbewusstsein”by Bischoff [22], the Body Awareness Questionnaire by Shields [23] and the „Fragebogen zur Wahrnehmung körperlicher Symptome”by Erdmann and Janke (not published). The “Fragebogen zum Köperbewusstsein”is a translation of the Body Consciousness Questionnaire by Miller et al. [24], but it is criticized for emphasising the emotional side too much. The Body Awareness Questionnaire investigates healthy non-emotional processes and reactions of the body (like circadian rhythms, changes of normal functions). The “Fragebogen zur Wahrnehmung körperlicher Symptome”investigates somatosensorical awareness. Due to contextual or statistical deficiencies of these three tools, Pöhlmann et al. designed a new test for the investigation of body awareness: the „Kurzer Fragebogen zur Eigenwahrnehmung des Körpers”(KEKS; short validated questionnaire of body self-awareness [25]. The KEKS is designed for the investigation of pure body awareness and assesses the distinction of awareness of body regions and bodily processes. It may differentiate between individuals with and without the ability to be aware of their own thoughts, emotions and needs.

However, the KEKS incorporates only certain regions of the body, e.g. backbone, shoulder blades, tongue, buttocks, eyelid, axilla—parts representing the awareness of “inner stability”, of “inner spaces”, and of the “boundary between interior and exterior”. Body parts like chest, abdomen, hand and leg are not mentioned. There is still a lack of a systematic self-assessment tool to scan the awareness of one’s body–including all body parts.

Furthermore, there are limitations with written or verbal questionnaires (reading and interpretation problems; predefined concept) [26]. To aid communication with patients, many health care professionals use anatomical maps, body charts or drawings, but without standardised scoring tools. Assessments using body charts have already been developed and used e.g. to investigate satisfaction with body parts [27, 28], to localise emotions within the body [29] or body charts to sign in pain localisation [30, 31]. Generally, the unconventional approach of colouring in the affected area in body charts is well accepted not only in children, but also in adults and may bring additional information through the creative expression of feelings. Hence, the Awareness-Body-Chart (ABC), a self-reporting assessment tool for the evaluation of body awareness was conceived. The aim of this study was to investigate the psychometric characteristics (reliability and validity) of this new instrument.

## Methods

### Sample

For this investigation physiotherapy students were chosen. Based on a standardized physical screening test on professional competences before admission for the degree program in Austria, one may presume these students to form a homogeneous group of adults with a sufficiently good and healthy state of body awareness. Students from the FH JOANNEUM University of Applied Sciences of Graz/Austria were recruited between April and September 2015. At the FH JOANNEUM University of Applied Sciences 172 were studying physiotherapy. For sample size considerations, we assumed a drop out of 20%. Therefore, we expected to investigate 138 students. Using this sample, a 95% confidence intervall for a Cronbach's alpha of 0.95, 0.85 and 0.75 would have a lower bound of 0.94, 0.81 and 0.69 respectively [32]. Although this sample size is sufficient for the estimation of the internal consistency, it gives only a first impression of the factorial structure of the questionnaire [33].

From the 172 full-time students (24.5% males), 106 students (25.5% males) agreed to participate: 79 females, age median 21 (*IQR* 19–23) years; 27 males, age median 22 (*IQR* 21–24) years. All participants filled in all forms and signed informed consent. The study was approved by the local ethics committee of the Medical University of Graz/Austria in compliance with the current revision of the Declaration of Helsinki, ICG Guideline for Good Clinical Practice and Current Regulations (EK-number: 27–245 ex 14/15).

### Instruments

**Fig 1:**
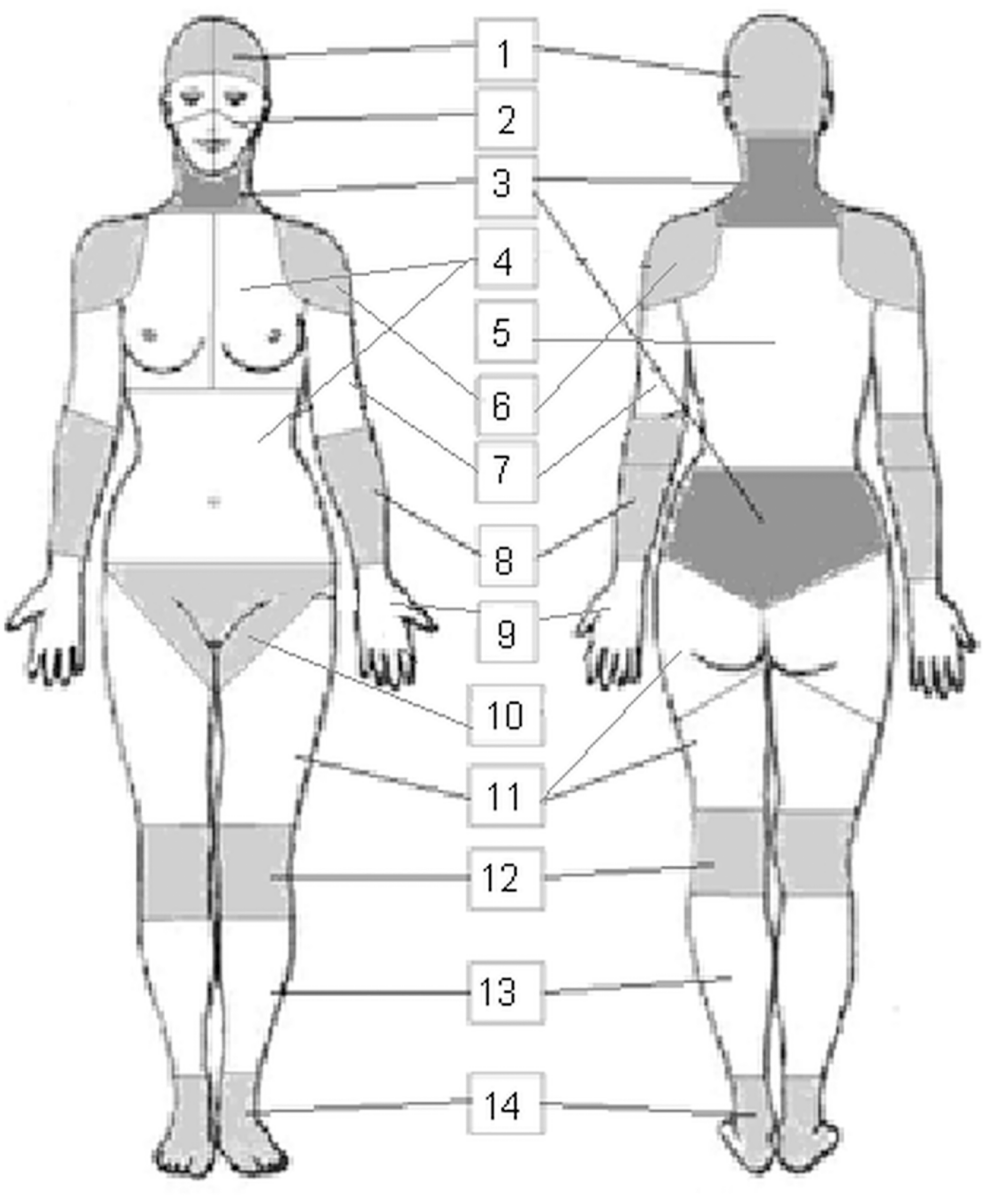
Illustration of the 51 regions and 14 body parts on the female body chart. 1 Cranium, 2 Face, 3 Cervical/lumbar region, 4 Chest/abdomen, 5 Back, 6 Shoulder, 7 Upper arm, 8 Lower arm/elbow, 9 Hand, 10 Genital area, 11 Thigh/hip, 12 Knee, 13 Lower leg, 14 Foot.

The ABC form consists of simple drafts of the front and back of the female and male body, respectively. The division in 51 regions was done according to anatomical structures. See Fig 1; original charts are without grey shadows and without the description of 14 body parts. In agreement with the intensity of their perception, subjects could use different coloured pencils to express their awareness of the different body regions. The colours corresponded with the level of awareness according to Pöhlmann, et al. [25]. The following colours were used: orange = “I can perceive with much detail”, yellow = “I can perceive distinctly”, green = “I can perceive”, blue = “I can perceive indistinctly”, black = “I cannot perceive”. To quantify the information, every region of the body was coded as an extra item and the data of the colours were transcribed: orange (= 5), yellow (= 4), green (= 3), blue (= 2), black (= 1). A red felt tip pen was at disposal to mark the localisation of possible pain awareness. Additionally, the pain intensity could be marked on a pain scale ranging from 0 to 100.

The KEKS consists of 20 items to be scored on a 5-point scale (“I cannot perceive” = 1 to “I can perceive with much detail” = 5). Two “falsehood-items”, left heart valve and cerebellum, which are not supposed to be perceived are included. On the basis of intercorrelation of the items (*r* = .62 - .69), a total score can also be used. The KEKS is a reliable (Cronbach’s alphas *α* = .71 - .93) and valid instrument.

To detect depressive mood, the German version of the Beck Depression Inventory II [34] (BDI-II) with 21 items and four answer options was included. The German version is widely used and shows good psychometric characteristics (in non-clinical samples internal consistency *α* ≥ .84 and retest reliability *r* ≥ .75) [35]. Additionally, descriptive personal data (sex, age, educational achievement, native language) and individual feedback from all participants were collected. The questionnaires were given in the following order: Personal data, ABC, KEKS, ABC (retest), ABC-Feedback, BDI-II. The procedure took 30 to 45 minutes.

### Statistics

The factor structure of the ABC was analysed using explorative principal component analysis. Cronbach’s alphas with 95% confidence intervals (95% CI) were calculated for each factor to assess the internal consistency. If required assumptions were met parametric statistics were used, otherwise non-parametric statistics were used. The test-retest reliability coefficient was calculated using Pearson correlation coefficient with 95% CI or by Spearman rank correlation coefficient with 95% CI. To investigate the validity, the association between the different questionnaires was calculated using Pearson correlation coefficient or Spearman rank correlations coefficient. Independent T-Tests and Mann-Whitney-U Tests were administered for the comparison of subgroups. Error probabilities below .05 were accepted to denote statistical significance. Psychometric analyses were performed using the statistical package SPSS version 22.0.

## Results

The highest (≥ 4.5) and the lowest (≤ 3.5) body awareness values were observed respectively in the regions of the hand and jaw and in the regions of the lower leg and upper arm dorsal. Overall only .6% of the regions had not been coloured. The most frequently missing regions were on the dorsal body chart. For 27 of the 51 regions of the body chart no missing data were found. 13 students used only two colours and only 7 students used the colour black (“I cannot perceive”).

A factor analysis with varimax rotation revealed 13 factors with an eigenvalue > 1 which explained 85.4% of the common variance. All items (body regions) assembled under a factor showed loadings higher than .40 (see Table 1) and, in almost every case, clearly corresponded and could be assigned to a physiological part of the body. The following regions had double loadings (higher than .40): left thigh ventral, right thigh ventral; throat, genital area, which means that they corresponded to more than one region. They were therefore adjudged after content and contextual analysis to a factor: left and right thigh ventral to “thigh/hip”; throat to “cervical/lumbar region”; the region “genital area” became an extra factor (body part). See the distribution of the 14 body-parts in the example of the female body drafts in Fig 1.

**Table 1:** The ABC-51 regions: Factor structure ^a^, factor loadings, medians (*IQR*), Cronbach’s alpha and test-retest reliability (*N* = 106).

Faktor I–XIV: Body part	Regions	Number of regions	Loadings	Median *(IQR)*	Cronbach’s α (95% *CI*)	Test-retest (95% *CI*)
I: Lower leg	Ri & le vent; ri & le dors	4	.843, - .810	3.5 (3.0–4.3)	.97 (.95 - .97)	.87 (.78 - .92)
II: Foot	Ri & le vent; ri & le dors	4	.866 - .826	4.9 (3.9–5.0)	.96 (.95 - .97)	.89 (.81 - .95)
III: Upper arm	Ri & le vent; ri & le dors	4	.766 - .733	3.3 (3.0–4.0)	.97 (.96 - .98)	.84 (.75 - .91)
IV: Lower arm/elbow	Ri & le vent; ri & le dors, elbow ri & le	6	.731 - .607	4.0 (3.3–4.3)	.93 (.90 - .95)	.83 (.71 - .92)
V: Shoulder	Ri & le vent; ri & le dors	4	.833 - .793	4.0 (3.0–4.5)	.94 (.91 - .95)	.85 (.77 - .90)
VI: Thigh/hip	Ri & le vent, ri & le dors; hip ri & le	6	.825 - .529	3.7 (3.3–4.3)	.91 (.89 - .94)	.86 (.78 - .92)
VII: Hand	Ri & le vent; ri & le dors	4	.869 - .735	5.0 (4.5–5.0)	.92 (.89 - .94)	.81 (.69 - .91)
VIII: Knee	Ri & le vent; ri & le dors	4	.838 - .667	4.0 (3.0–4.5)	.90 (.86 - .93)	.76 (.65 - .86)
IX: Face	Ri & le eye; ri & le jaw	4	.834 - .739	4.5 (4.0–5.0)	.91 (.87 - .93)	.90 (.85 - .95)
X: Cranium	Ri & le front; head dors	3	.926 - .605	4.0 (3.0–4.7)	.90 (.86 - .93)	.83 (.73 - .89)
XI: Chest/abdomen	Ri & le chest, abdomen	3	.769 - .540	4.0 (3.3.-4.3)	.84 (.77 - .88)	.79 (.87 - .89)
XII: Cervical/lumbar region	Neck, throat, lumbar region	3	.729 - .445	4.0 (3.6–4.4)	.64 (.50 - .74)	.86 (.80 - .91)
XIII: Back	Back	1	-.527	4.0 (3.0–5.0)	-	.71 (.60 - .82)
XIV: Genital area	Genital area	1	^b^	4.0 (4.0–5.0)	^-^	.83 (.69 - .92)

^a^ Exploratory factor analysis with varimax rotation;

^b^ no loading, because this factor was added later.

*IQR* = Interquartilrange; 95% *CI* = 95% confidence interval; ri = right; le = left; dors = dorsal; ventr = ventral.

Table 1 showed for each body part both an acceptable to high internal consistency, determined by Cronbach’s alpha (*α* = .64 - .97) and an acceptable to high test-retest reliability (using an interval of *ρ* = .71 - .90). The analysis of the total score (mean 3.9, *SD* .56) showed a Cronbach’s alpha of *α* = .96 (95% *CI* .95 - .97) and in the test-retest reliability of *r* = .96 (95% *CI* .93 - .97). The intercorrelation matrix also underlined high values. Only a few body parts showed non-significant values with Spearman’s *ρ* < .160 (see Table 2).

**Table 2:** Spearman Intercorrelation of the body parts (*N* = 106).

	Total score	1	2	3	4	5	6	7	8	9	10	11	12	13	14
1 Cranium	.480**														
2 Face	.616**	.443**													
3 Cervical/lumbar region	.612**	.460**	.409**												
4 Chest/ abdomen	.675**	.286**	.476**	.443**											
5 Back	.414**	.316**	.151	.340**	.393**										
6 Shoulder	.645**	.221*	.287**	.415**	.428**	.323**									
7 Upper arm	.747**	.355**	.452**	.481**	.490**	.295**	.552**								
8 Lower arm/elbow	.799**	.400**	.440**	.443**	.476**	.238*	.525**	.691**							
9 Hand	.515**	.168	.308**	.188	.243*	.138	.265**	.355**	.460**						
10 Genital area	.538**	.082	.258**	.272**	.488**	.157	.271**	.402**	.385**	.298**					
11 Thigh/hip	.712**	.238*	.333**	.349**	.449**	.299**	.345**	.514**	.489**	.266**	.429**				
12 Knee	.630**	.247*	.348**	.342**	.314**	.328**	.421**	.277**	.421**	.331**	.385**	.385**			
13 Lower leg	.744**	.314**	.426**	.377**	.487**	.383**	.397**	.540**	.597**	.260**	.322**	.582**	.427**		
14 Foot	.543**	.081	.304**	.181	.356**	.054	.343**	.230*	.306**	.410**	.472**	.370**	.329**	.323**	

**. Correlation is significant at the .01 level (2-tailed).

*. Correlation is significant at the .05 level (2-tailed).

Significant correlations are given with bold characters.

The mean KEKS sum score was 3.4 (*SD* 1.3). The highest awareness in the KEKS was found in the tongue with 5.0 (*IQR* 4.0–5.0) and toes 4.0 (*IQR* 4.0–5.0). The lowest values (excluding the two falsehood-items cerebellum and left heart valve) were hairline 3.0 (*IQR* 2.0–4.0) and tailbone 3.0 (*IQR* 2.0–4.0). The ABC total score correlated with the KEKS-score with *r* = .66 (*p* < 0.001).

Eighty-seven of the participants showed a BDI-II value lower than 9. Seventeen participants had a BDI-II ranging from 9–13 points which indicated a minimal depression. Two participants had a score corresponding with a mild depression (14–19 points). The BDI-II score correlated highly significantly with the ABC total score (*ρ* = -.41; *p* < .001), shoulder (*ρ* = -.35; *p* < .001), thigh/hip (*ρ* = -.41; *p* < .001), chest/abdomen (*ρ* = -.35; *p* < .001), and back (*ρ* = -.34; *p* < .001).

Participants who had scored falsehood items in the KEKS (*n* = 29) had higher body awareness in the ABC in hand (5.0, *IQR* 4.3–5.0 versus 4.5, *IQR* 4.0–5.0; *p* = .015), in cranium (4.3, *IQR* 4.0–5.0 versus 3.7, *IQR* 3.0–4.7; *p* = .011), in chest/abdomen (4.3, *IQR* 3.7–5.0 versus 3.7, *IQR* 3.3–4.3; *p* = .008), and in back (4.0, *IQR* 4.0–5.0 versus 4.0, *IQR* 3.0–5.0; *p* = .018), but not in the total score. Furthermore, there was no difference between those two subsamples concerning the BDI-II.

On the ABC no significant differences were found between females and males, except that thigh/hip showed significant higher values for males (3.7, *IQR* 3.2–3.3 versus 4.2, *IQR* 3.7–4.7; *p* = .036). Also, the comparison between subjects with “no actual pain” (*n* = 43) and “with actual pains” (*n* = 63) showed no significant differences except for the body awareness in genital area with higher awareness in subjects without pain (“no pain” 5.0, *IQR* 4.0–5.0 and “pain” 4.0, *IQR* 3.0–5.0; *p* = .038). The ABC distinguished between students who had just entered into the physiotherapy course (*n* = 47) and their advanced colleagues (*n* = 59) on the total score (new students mean = 3.8 (*SD* .6) versus advanced students 4.1 (*SD* .5); *p* = .005. New students showed lower scores on hand, knee, face, lower arm/elbow, thigh/hip, and genital area.

## Discussion

In the last years, many scientists have used the word interoception as an umbrella concept for a multi-sensory, multimodal integrated percept of the body state, inclusive definitions of proprioception, interoception and exteroception, where all that matters is the phenomenological experience and not which type of receptors are involved in creating that experience [3]. This expresses what we define with the global description of body awareness.

To explore the interactions of body and mind in its subjective, objective and metacognitive dimensions, new assessments of body perception are demanded [12]. In order to counteract the limitations of verbal questionnaires (reading/interpretation problems) [36]the ABC was designed to assess the intensity of body awareness. In this study, the psychometric features of the ABC were tested.

Fourteen factors of the ABC with clear assignments to body parts could be categorized: Cranium, face, cervical/lumbar region, chest/abdomen, back, shoulder, upper arm, lower arm/elbow, hand, genital area, thigh/hip, knee, lower leg, and foot. Each body part showed acceptable to high internal consistency. Each of the 14 factors can therefore be analysed separately. The lowest Cronbach’s alpha was shown in cervical/lumbar region which is plausible because neck, throat, and lumbar region are not contiguous items. In special assessments focussed on these regions they may be analysed distinctly.

The highest awareness was found for hand, face, and foot, which is in accordance with the cortical homunculus of the primary somatosensory cortex [37, 38]. This main sensory receptive area for the sense of touch is known for a large representation of face, hand, and sole of foot. The test-retest reliability was also acceptable to high for all body parts. Knee, chest/abdomen, and back showed the lowest retest reliability. We suppose that body awareness in these parts changes quickly in the sitting position. Further investigation is warranted to affirm this assumption. An alternative explanation of the low retest reliability of chest/abdomen could be that chest as well as abdomen would typically change depending on emotional state (i.e. autonomic activity associated with various emotional states). Thus, an alternative explanation could be mood or emotional state influencing the awareness of chest/abdomen.

The correlation between the total score and the single parts indicated that the total score could be used for global descriptions of body awareness measured by the ABC. Cranium and back showed the lowest correlation values with the total score. This is consistent with the strong influence of visual control on body awareness known from literature [39, 40]. As the ABC is a test on the basis of optical reproduction, one can assume that body regions that are difficult to see by oneself (as cranium and back) might have lower correlation values as they are out of optical control.

The relation of the total score of the ABC which delivers a systematically global scan of the body top down and the KEKS which assess specific details of the body was reasonably high to confirm construct validity. Correlations with other instruments (KEKS and BDI-II) showed that the ABC had satisfactory validity. It is generally stated in psychological research that bodily symptoms, as well as mood changes are often a consequence of alteration of awareness and/or vice versa [7, 41]. Our findings of a high correlation of the ABC and BDI-II are in line with this assumption. Nevertheless, it must be noted that the tested cohort generally consisted of psychologically healthy people and therefore depressive symptoms measured with the BDI-II were minimal. Conclusions for people with psychiatric disorders can therefore not be drawn from the present data. Even so, the ABC represents a practical tool to use for further examinations on the relationship between actual mood and the awareness of body parts. Further research is warranted with different samples and additional psychological tests. It may add important information in differentiating between anxiety and depression and other mood shifts which may develop different patterns of awareness.

Of particular interest was that with regard to the KEKS questionnaire, 29 subjects gave points for the falsehood-items, i.e. for the awareness of the left heart valve and for the cerebellum. Typically, these regions are not supposed to be sensed. Additional correlation of the falsehood-items with the BDI-II items demonstrated no link. An explanation of the finding might be the so-called “medical students’ disease” [42], an incorrect interpretation of certain physical symptoms and a normal phenomenon especially in the early phase of medical training. It is acceptable that this is also the case in students of physiotherapy. However, participants who had scored falsehood items in the KEKS showed no difference in the ABC total score, but higher body awareness in the ABC in hand, cranium, chest/abdomen, and in back. These findings suggest that these regions should be carefully investigated with regard to specific problems. Furthermore, another assumption of the „erroneously”scored regions leads us to the widespread phenomenon of the “medically unexplained symptoms”. In a study of Reid et al. [43], it can be read that these unexplained symptoms are mostly found in the regions of the abdomen, chest, back and head. A connection with the present data, which showed increased correlation value in cranium, face, chest/abdomen and back, may be assumed. This means that especially with suspicious ABC values in these body parts, investigators should not only think of local problems in this body region, but consider stressful life events, anxiety and other sources of “medically unexplained symptoms”.

Additionally, indications of external validity were found by the comparisons of subgroups. As physiotherapy education implies an intensive self-awareness training, we supposed that physiotherapy students would have higher body awareness than other young people. Indeed, the new students had significantly lower values of body awareness than more advanced students. Furthermore, contrary to what could be expected according to wide spread opinions, no sex-related differences in body awareness were observed except for thigh/hip.

In a next step, we distinguished between students who had marked actual pain perception on the ABC and those who did not. Our results revealed no differences between these groups, with one exception: there was higher body awareness of the genital area in the group with no actual pain. This finding is supported by literature. In persons without explicit pain catastrophizing tendencies, no relation was found between the level of pain and the body awareness [44]. Additional research is warranted to investigate the relation between the perception of pain and body awareness in different samples [36, 44–48].

The findings of the present study need to be interpreted with caution due to methodological limitations: firstly, the use of a self-report measurement even if the reliability and validity is acceptable, and the absence of an objective measurement of body awareness. Secondly, the sample consisted of a non-clinical group of physiotherapy students and did not include parameters such as socio-economic status. Thirdly, we had only a small sample size of 106 investigated students. De Winter et al. [33] reported differences in the recommended minimum sample size for factor analysis ranging from 50 to 1000. They have shown that small sample sizes are sufficient for high factor loadings resulting in only 47 subjects needed if 8 factors are extracted out of 48 items with factor loadings of 0.8, with even smaller required sample sizes for factor loadings of 0.9. In our study 21 out of 51 items showed factor loadings of > 0.8 and 24 between 0.6 and 0.8. Nevertheless, the factor structure has to be validated in a bigger sample.

Besides the score, other additional information during the assessment of the ABC questionnaire can be observed. The pressure on the pencil and the accuracy in filling in the forms, the time used to complete the questionnaire, etc. may give some additional information of relevance for clinical practice. In addition to the subject of investigation, the feedback of participants indicated that the ABC not only assessed the momentary state of awareness, but also helped to modulate the awareness. The finished coloring represented an interesting impression about one’s own mirror image, which stimulates reflecting about the momentary state of self-awareness. This opens the therapeutic field for the implementation of the ABC-form as a tool for self-control. From literature we know that the more subjects get used to controlling and modulating their self-awareness in exercise situations, the more they can use stress coping strategies in the presence of stressful environments [7, 49–53]. For people with either psychiatric or somatic problems, self-assessments of their body awareness and reflecting on the bodily experience–from the perception of body parts and the combined affects and emotions, is often a first step in getting in touch with their feelings [54]. Understanding one's emotions and needs through the awareness of body parts can be the base for self-confidence, trust in one-self and the ability to take care of oneself and personal needs both physically and mentally [5]. Until now the therapeutic effect of the implementation of the ABC has not been examined, however.

The ABC represents a new non-verbal measure of body experience and may relate differently to other subjective, objective and metacognitive aspects of interoception [12], interoception. Further implementing of the ABC in clinical work and in research will give insights of the value of the ABC and information of body awareness within different clinical and non-clinical groups.

### Conclusion

Despite the importance of body awareness for health and well-being there is still a lack of valid nonverbal assessment tools to scan proper body awareness [26]. We designed a simple nonverbal test, the Awareness-Body-Chart (the ABC) to assess body awareness by colouring the different areas from the top of the head to the feet. The newly developed ABC is an easy-to-use tool, at low cost, not invasive and independent of verbal skills. The ABC exhibits good psychometric properties with acceptable to high internal consistency, an acceptable to high test-retest reliability and satisfactory validity. It is an assessment tool for clinical practice as well as for scientific research. It opens new insights into body awareness patterns in different subgroups.
